# Research advance in correlation between childhood asthma and gut microbiota

**DOI:** 10.3389/fcimb.2025.1649180

**Published:** 2025-10-06

**Authors:** Yucong Ma, Haoqi Zheng, Hai Tian, Jingjing Cui, Li Liu

**Affiliations:** ^1^ Department of Pediatric Respiratory, Children’s Medical Center, The First Hospital of Jilin University, Changchun, China; ^2^ Department of Rheumatology and Clinical Immunology, Tianjin Children’s Hospital, Tianjin, China; ^3^ Department of Pediatrics, Central Hospital of Songyuan City, Songyuan, China

**Keywords:** gut microbiota, asthma, immune modulation, gut-lung axis, pathogenesis

## Abstract

Asthma remains a highly prevalent and heterogeneous chronic respiratory disease in the pediatric population. Accumulating evidence has established a critical role of the gut–lung axis in the pathogenesis of asthma. Specifically, gut microbiota constituents such as Lactobacillus and Bifidobacterium species have been closely associated with childhood asthma. Current intervention strategies targeting the gut microbiota show considerable promise. Probiotic supplementation and short-chain fatty acids (SCFAs) have demonstrated the ability to restore microbial homeostasis and suppress type 2 inflammation. Additionally, phytogenic compounds exhibit potential in reshaping the gut microbial composition and ameliorating asthma symptoms. This review synthesizes clinical and mechanistic evidence from both human and animal studies, highlighting the translational potential of microbiota-targeted therapies. Future research should prioritize the development of personalized microbiota-based interventions to improve clinical outcomes in children with asthma.

## 1 Introduction

Asthma was identified by the Global Burden of Disease study as being the most globally prevalent chronic respiratory disease, affecting more than 300 million people, and the figure would go up to 400 million by 2025 (Dharmage et al., 2019; Serebrisky and Wiznia, 2019). Epidemiological surveys show that the prevalence of asthma in children is much higher than that of adults, and the global incidence of childhood asthma has risen from 11.1% to 13.2% in the past 10 years (Licari et al., 2018). Although hospitalizations and deaths from asthma have declined in some countries, childhood asthma still places an unacceptable burden on the health care system and society.

Asthma is a chronic airway disease characterized by airway inflammation, involving a variety of immune cells including lymphocytes, mast cells, basophils, eosinophils, and neutrophils. The pathogenesis of asthma is complex and related to allergic factors, family history, lifestyle and environmental factors. Furthermore, the immune system plays a central role in the pathophysiology of asthma, and recent research had emphasized the importance of the microbiome in the development of immune responses (Zheng et al., 2020). In recent years, increasing evidence has revealed that the occurrence of allergic diseases is related to the microbial composition of the body (Plunkett and Nagler, 2017; Aguilera et al., 2020; Navanandan et al., 2021; Sarikloglou et al., 2023). Gut microbiota and their metabolites play a crucial role in the development and regulation of the human immune system, capable of modulating distant organs.

The human microbiome has the functions of regulating host metabolism, maintaining immune system homeostasis and preventing pathogen invasion. The microbiome is distributed in all parts of the body, with the gastrointestinal tract and respiratory system being the primary residence. Under conditions of homeostasis, a healthy gut microbiota can effectively prevent pathogenic infections and reduce the occurrence of inflammation. Conversely, dysregulation of the gut microbiota may lead to altered immune responses and chronic inflammatory respiratory diseases, especially asthma. In fecal samples from newborns at high risk for developing asthma, a reduction in the numbers of *Lachnospira*, *Veillonella*, *Fecalibacterium*, and *Rothia* strains has been observed (Arrieta et al., . 2015). And, the mutual influence of microbial composition and function between the respiratory tract and the gut is referred to as the “gut-lung axis” (Belkaid and Hand, 2014).

In recent years, due to the association between the gut microbiome and asthma, it has gained increasing attention as a key regulator of host health. This review primarily summarizes the current understanding of the immunological mechanisms underlying asthma, as well as the role of the gut microbiota and the gut-lung axis in asthma. Additionally, we explore the possibility of developing asthma prevention strategies by regulating the microbiome and optimizing its treatment options. To investigate the association between the gut microbiome and asthma, we conducted a systematic search of the Web of Science and PubMed databases for original research articles, reviews, and systematic reviews published between January 2012 and May 2025. The search strategy incorporated the following keyword combinations: (‘gut microbiota’ OR ‘gut microbiome’ OR ‘gut microbial metabolites’ OR ‘gut-lung axis’) AND (‘allergic asthma’ OR ‘asthma’). Our search was limited to English-language literature. Initial screening was performed using the respective databases’ built-in search engines. Studies were included based on their relevance to gut-lung communication mechanisms, the effects of microbial dysbiosis, and the role of gut microbiota in asthma pathogenesis.

## 2 The immunopathogenesis of asthma

Asthma is a heterogeneous disease that encompasses various phenotypes and endotypes, with T helper cell type (Th)-2 high asthma, also known as allergic asthma, being one of the most common phenotypes. Th2 inflammatory response is one of the mechanisms driving the development of allergic asthma, in which the imbalance of T lymphocyte subsets (Th1/Th2) is the key to the development of asthma.

**Figure 1:**
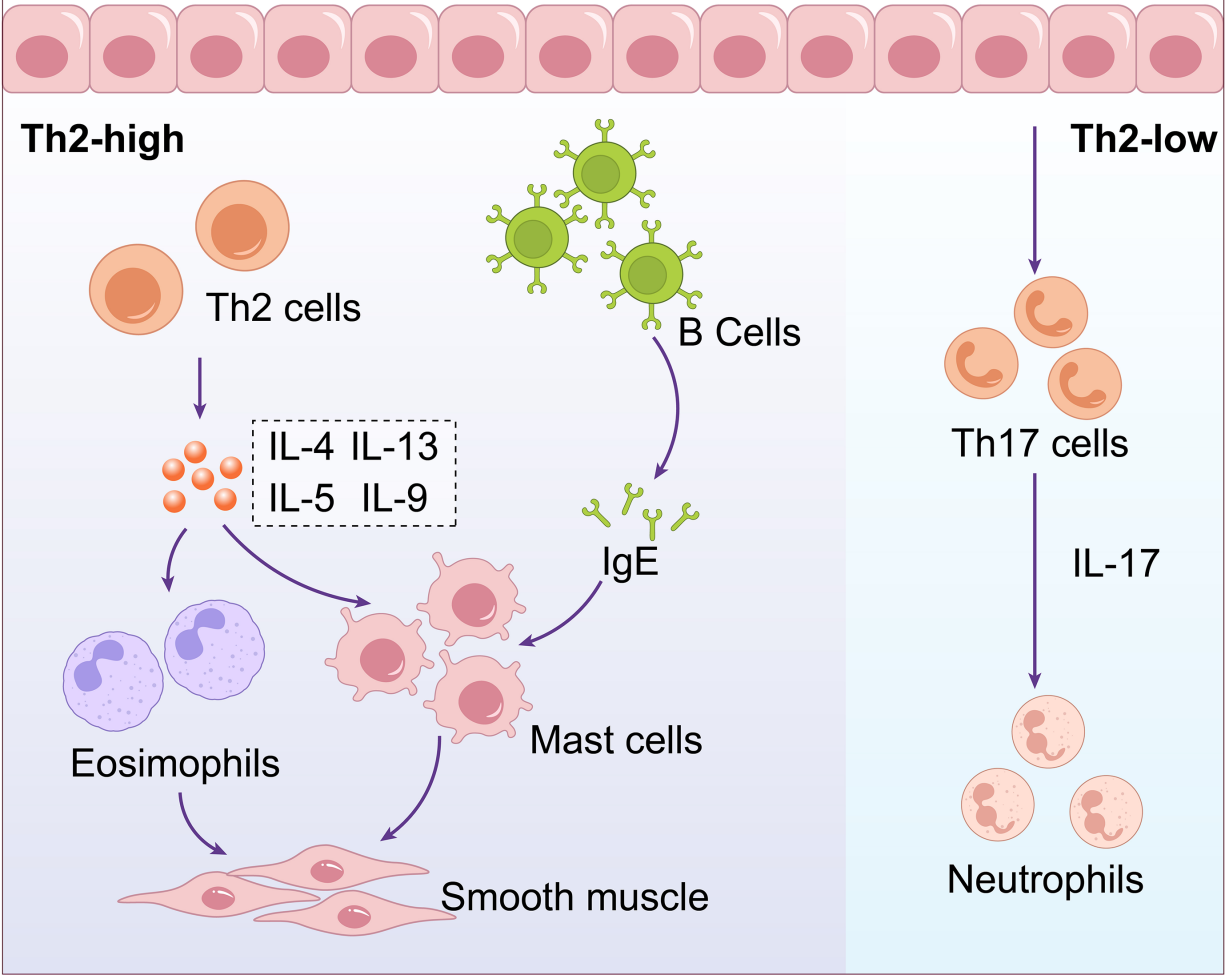
Schematic diagram of asthma endotypes and their distinct inflammatory pathways. Asthma can be categorized into two main endotypes: Th2-high involves Th2 cells producing IL-4, IL-5, IL-9, and IL-13, affecting B cells,eosinophils, mast cells, and smooth muscle. Th2-low involves Th17 cells producing IL-17,influencing neutrophils.

Upon stimulation by allergens, airway epithelial cells activate dendritic cells to present antigens, which subsequently drive the differentiation of naive T cells into effector Th2 cells. Th2 cells participate in eosinophilic inflammation through the secretion of Interleukin (IL)-4, IL-5, and IL-13. IL-4 promotes the differentiation of Th0 cells into Th2 cells and the production of immunoglobulin E (IgE) by B lymphocytes, IL-5 promotes the maturation and activation of eosinophils, IL-13 induces the production of IgE by B lymphocytes, facilitates the migration of eosinophils to the airways, promotes fibroblast proliferation and collagen synthesis, and can also induce airway smooth muscle cell contraction, leading to increased airway responsiveness. Additionally, under the stimulation of IL-4, B cells differentiate into plasma cells and produce specific IgE antibodies. These IgE antibodies bind to receptors on the cell surface, such as mast cells and basophils, and mediate the release of mediators such as histamine, leukotrienes, and platelet activating factor (Brooks et al., 2013; Foster et al., 2017; Cao et al., 2024). This is a primary mechanism that contributes to the development of allergic asthma. (**Figure 1**. Schematic Diagram of Asthma Endotypes and Their Distinct Inflammatory Pathways).

Additionally, there exists Th2-low asthma, also known as non-eosinophilic asthma, which lacks Th2 biomarkers or elevated eosinophils. This type of asthma is primarily associated with aberrant Th17 or Th1 cell immune responses. In this process, an imbalance between Tregs and Th17 cells is a crucial factor in the disease’s pathogenesis. Under the influence of various pro-inflammatory cytokines (such as IL-6, IL-1β), Cluster of Differentiation 4 (CD4) + T cells develop into Th17 cells. These Th17 cells release IL-17, which recruits neutrophils, leading to exacerbated airway inflammation (Hudey et al., 2020). Enzymes released by neutrophils, such as neutrophil elastase, can damage the airway epithelial barrier, resulting in persistent inflammation and structural changes in the airways (Varricchi et al., 2022) (**Figure 1**. Schematic Diagram of Asthma Endotypes and Their Distinct Inflammatory Pathways).

## 3 The gut-lung axis and asthma

### 3.1 Gut microbiota and asthma

The gut is the most densely colonized site in the human body, harboring over 10^14^ bacterial cells, comprising more than 1,000 different species, which together form one of the most complex and diverse ecosystems (Lunjani et al., 2018). The phyla *Firmicutes, Bacteroidetes, Actinobacteria* and *Clostridia* are the major constituents of this community. The development and maintenance of a healthy gut microbiota are critical for human health, as these microorganisms participate in numerous physiological functions, including the enzymatic breakdown of complex carbohydrates, vitamin production, ion absorption, defense against pathogens, tissue development, enhancement of immune function, and food fermentation (Hillman et al., 2017). The ratio and composition of the gut microbiota are dynamic, research indicates that the early-life gut microbiota develops through three distinct stages: a developmental phase (3–14 months), dominated by *Bifidobacterium*; a transitional phase (15-30months), characterized by increasing abundances of *Proteobacteria* and *Bacteroidetes*; and a stable phase (≥31 months), during which *Firmicutes* become the predominant bacterial phylum (Mogoş et al., 2025).Biodiversity plays a crucial role in the gut microenvironment. Loss of diversity is one of the reasons for the recent increase in asthma prevalence (Azad and Kozyrskyj, 2012). Studies have shown that breastfeeding can effectively prevent childhood asthma and allergic diseases, which may be related to the richer gut microbiota in breastfed infants (Oddy, 2017; Wang et al., 2018). *Akay* et al. found that the detection rate of *Bifidobacterium longum* was lower in children with asthma compared to healthy children (Akay et al., 2014). In children, oral administration of Lactobacillus and Bifidobacterium can help relieve asthma symptoms (Yang et al., 2023). Mahdavinia et al. found that the abundance of *Prevotella copri*, *Bifidobacterium breve*, and *Bifidobacterium catenulatum* was reduced in children with asthma (Mahdavinia et al., 2023). In murine models of asthma, early-life exposure to cefixime has been demonstrated to induce significant intestinal dysbiosis, characterized by an enrichment of genera such as Isprevalenzia, Brauneria, and Marvinbryantia. These specific microbial alterations show a positive correlation with pulmonary eosinophil infiltration and Th2 cell activation. Conversely, antibiotic-induced dysbiosis reduces the production of short-chain fatty acids (SCFAs), thereby impairing intestinal epithelial barrier function and weakening regulatory T cell-mediated immunosuppression. Furthermore, this microbial imbalance disrupts pulmonary lipid metabolism, which exacerbates type 2 allergic airway inflammation by increasing airway epithelial permeability and promoting mast cell degranulation. These findings suggest that changes in the abundance of specific gut microbial genera and particular dysbiotic states are closely associated with the pathogenesis of asthma (Kong et al., 2024).

### 3.2 Lung microbiota and asthma

The lungs were once mistakenly believed to be sterile. With advances in testing technologies, particularly the use of 16S rRNA and metagenomic sequencing, the presence of a lung microbiome in healthy individuals has been confirmed. The biomass of the lung microbiome is significantly lower than that of the gut microbiota, with approximately 10–100 bacteria per 1000 human cells (Kong et al., 2023). The biomass in the respiratory tract decreases from the upper to the lower respiratory tract, The most common communities include Actinobacteria, Firmicutes, Proteobacteria, and Bacteroidetes. Moreover, a study had found that when lung diseases such as asthma occur, there is a certain degree of ecological imbalance in the lung microbiota (Durack et al., 2017). Notably, Wang et al. explored the sputum microbiome of asthmatic patients. patients and discovered a link between lower Granulicatella abundance and asthma, as well as an increase in Streptococcus in the respiratory microbiome of asthma patients (Wang et al., 2018). Thorsen et al. conducted a prospective cohort study that highlighted the link between the respiratory microbiome and the respiratory immune system, offering new evidence for the impact of early microbial communities and their interactions with immunological components on asthma risk (Thorsen et al., 2019).

### 3.3 Gut–lung axis

The gut-lung axis plays a major role in respiratory system dysfunction because it creates a bidirectional conduit for the transfer of internal and external variables, forming a signaling system that can alter systemic function and reactions (Enaud et al., 2020). The gut-lung connection notion is based on the finding that alterations in the gut milieu affect many lung disorders, and vice versa. Such as chronic respiratory diseases, including asthma and chronic obstructive pulmonary disease, which are often associated with gastrointestinal diseases or symptoms (Vutcovici et al., 2016; Labarca et al., 2019). Individuals who suffer from gastrointestinal disorders, such as gastric reflux and inflammatory bowel disease, are more likely to experience lung disease morbidity and pulmonary dysfunction (Jové Blanco et al., 2020; Lu et al., 2023). These correlations point to an important exchange of information between the lungs and the gut. Although the gut is anatomically isolated from the respiratory tract, but epithelial cells of both the gastrointestinal and respiratory tract are known to develop from common embryonic structures. The interaction between these two mucosal regions is believed to be caused by the human microbiome, which also plays a role in homeostasis and illness. It is crucial to further emphasize the fact that significant differences exist between the lung and gut microbiomes in both composition and density. The lungs, being a relatively low-biomass organ continuously exposed to the external environment, host a core microbiota that, while also predominantly composed of Firmicutes and Bacteroidota, demonstrates substantially lower species diversity and abundance of dominant genera compared to the gut. This disparity stems from the distinctly different physicochemical properties of the two organ’s microenvironments; for instance, the hypoxic and nutrient-rich milieu of the gut stands in stark contrast to the oxygen-rich environment of the lungs. Changes in microbial composition or/and diversity can directly affect not only the colonized organs themselves, but also the distant organs and systems (Feng et al., 2018). Thus, increasing evidence highlights the relationship and crosstalk between the gut and the lung, namely the gut–lung axis (Frati et al., 2018).

The core mechanism of the gut-lung axis fundamentally constitutes a functional bidirectional communication network between these two distant organs, primarily mediated through immune and metabolic pathways. Recent investigations focusing on the respiratory immune system have further elucidated that gut microbiota plays a pivotal regulatory role within this gut-lung axis (He et al., 2017). For example, mice raised in axenic or germ-free conditions exhibit stronger allergic responses to allergen challenge due to the lack of gut microbiota (Liu et al., 2021). Interestingly, the development of respiratory disease-associated immune responses is reversible and controllable through targeted modulation of specific microbiota or application of probiotics (Li et al., 2020a). Studies on mice and humans showed similar outcomes, suggesting that oral *L. rhamnosus*, *L. casei*, and *Bifidobacterium brevis* may prevent and treat allergies and asthma (Chunxi et al., 2020). Studies have demonstrated that when intestinal or pulmonary dysbiosis occurs, immune cells such as ILC2s can migrate between the lung and gut via the bloodstream, releasing excessive amounts of inflammatory mediators, thereby altering the pulmonary microenvironment and influencing the type and intensity of immune responses (Shi et al., 2021). The aforementioned related immune and metabolic mechanisms will be elaborated in detail in Section “4 The Main Mechanisms of Gut Microbiota in Regulating the Occurrence and Development of Asthma”.

Regarding the question of whether microbes themselves can translocate bidirectionally between the lungs and intestines via the bloodstream, current evidence indicates that this is a limited yet demonstrably existent pathway. Human blood was previously considered a sterile component, and the presence of live microorganisms in the bloodstream could lead to sepsis. However, Potgieter et al. introduced the term “atopobiosis” to describe the presence of microbes in the blood. This term suggests that translocated microbes may lie dormant in the circulation in a harmless state and could potentially be stimulated under favorable conditions to initiate immune responses (Potgieter et al., 2015). Several studies on the blood bacteriome of healthy individuals have revealed that the composition at the phylum level includes *Proteobacteria*, *Actinobacteria*, and *Firmicutes*. These three phyla account for over 70% of the blood bacteriome, while *Bacteroidota*, *Fusobacteriota*, *Cyanobacteria*, *Verrucomicrobiota*, and *Acidobacteria* are minor constituents (Kajihara et al., 2019; Emery et al., 2020; Suparan et al., 2022). Asthmatic patients exhibit an increase in Bacteroidota within their blood’s dysbiotic microbial signature. A potential mechanism is that during airway inflammation, the lung bacteriome, which can be predominantly composed of Bacteroidota, translocates into the circulatory system (Khan et al., 2025). Research indicates that ARDS induced by sepsis and other critical illnesses damages alveolar epithelial cells and pulmonary capillary endothelial cells, increasing the permeability of the alveolar-capillary membrane. These severe conditions also trigger a massive release of inflammatory mediators, which heightens intestinal permeability. This breach allows gut-derived bacteria and endotoxins to translocate via the circulatory system to the lungs, leading to an enrichment of gut-associated bacteria (such as Bacteroides spp. and Enterobacteriaceae) in the pulmonary environment. This process disrupts the native lung microbiome and exacerbates the progression of ARDS (Mukherjee and Hanidziar, 2018; Yang et al., 2025). Conversely, gut microbiota dysbiosis compromises intestinal barrier integrity, increases intestinal permeability, and promotes the translocation of bacteria and endotoxins such as lipopolysaccharide (LPS) into the systemic circulation, thereby triggering chronic pulmonary inflammation (Schoultz and Keita, 2020; Chen et al., 2025).

Therefore, beyond the well-established immune and metabolic pathways, the direct translocation of microbes via the bloodstream may represent a hitherto understudied yet tangible dimension of the gut-lung axis. While this bidirectional material exchange is likely minimal under homeostatic conditions, it can be substantially amplified during states of inflammation, infection, or barrier dysfunction, thereby playing a critical role in the pathogenesis and exacerbation of diseases such as asthma. We anticipate that further animal studies will provide definitive evidence for the direct hematogenous migration of viable bacteria between the gut and the lungs.

## 4 The main mechanisms of gut microbiota regulating the occurrence and development of asthma

### 4.1 Immune system imbalance

#### 4.1.1 Regulating the Th1/Th2 balance

The uterus is where the immune system develops, and during pregnancy, the mother’s surroundings encourage the fetal immune system to mount a Th2 response. After birth, when the body is exposed to or infected with microorganisms, Th1-type cytokines such as IL-2 and interferon-gamma (IFN-γ) can be induced, leading to the differentiation of Th0 cells towards Th1 cells that combat infection, thus achieving Th1/Th2 immune balance. If this transition is delayed or impaired in the early postpartum period, the risk of atopic diseases increases[20]. Therefore, the imbalance of T lymphocyte subsets (Th1/Th2) is an important mechanism in the development of asthma. Under healthy conditions, the gut microbiota maintains Th1/Th2 equilibrium by orchestrating dendritic cell (DC) function, thereby suppressing asthma-associated inflammation. In contrast, gut dysbiosis disrupts this equilibrium, driving hyperreactivity of the Th2 immune response, which leads to elevated IL-4 levels and eosinophil infiltration. The specific mechanisms involved are as follows:

##### 4.1.1.1 Beneficial bacteria promote Th1 cell differentiation and antagonize Th2-mediated inflammatory responses

Dominant beneficial gut bacteria, such as Bifidobacterium and Lactobacillus genera, can engage with Toll-like receptor 2 (TLR2) on the surface of intestinal dendritic cells (DCs) via their surface-active components (e.g., Bacteroides fragilis polysaccharide A, PSA). This interaction induces DCs to secrete interleukin-12 (IL-12). IL-12 further activates the STAT4 signaling pathway within T cells, driving the differentiation of naive CD4^+^ T cells into T helper 1 (Th1) cells, which subsequently secrete interferon-gamma (IFN-γ). IFN-γ directly suppresses the activation and proliferation of Th2 cells, thereby reducing the secretion of Th2-type cytokines such as IL-4 and IL-5. Concurrently, it inhibits the differentiation of B cells into IgE-synthesizing plasma cells, indirectly mitigating the chemotaxis and activation of eosinophils (Idrees et al., 2022). *Kei E* et al. found that supplementation with *L. johnsonii* in mice significantly reduced the Th2 response and decreased both allergy and airway hyperresponsiveness (Fujimura et al., 2014). In mice models of asthma, *L.reuteri* alleviates airway inflammation, reduces total IgE levels, and decreases Th2-associated pro-inflammatory cytokines (Li et al., 2020b).

##### 4.1.1.2 Gut dysbiosis induces overactivation of Th2 immunity

When the structure of the gut microbiota is disrupted (e.g., increased abundance of potential pathogens such as Streptococcus in children with asthma) (Pattaroni et al., 2025), bacterial cell wall components (e.g., lipopolysaccharide, LPS) or toxins alter the antigen-presenting bias of dendritic cells (DCs). Specifically, DCs upregulate the expression of costimulatory molecules CD80/CD86 and cease producing IL-12, instead secreting IL-4, which drives the differentiation of naive T cells into Th2 cells. Mature Th2 cells migrate to the airways via the bloodstream and, upon re-exposure to allergens, secrete large quantities of IL-4 (Zhuang et al., 2016; Hammad and Lambrecht, 2021).

#### 4.1.2 Regulating Th17/Tregs cell balance

Treg cells serve as the pivotal effector cells through which the gut microbiota orchestrates the suppression of asthmatic inflammation. Tregs and their cytokines have anti-inflammatory properties, while Th17 cells and IL-17 can trigger inflammatory responses. Once this balance is disrupted, it can lead to allergic diseases. Research has indicated that individuals with asthma exhibit an imbalance in the peripheral blood Th17/Tregs ratio, with elevated Th17 and reduced Tregs (Shi et al., 2021).

PSA from *Bacteroides fragilis* can induce the differentiation of CD4+ T cells in the intestinal lamina propria and in the circulation into Treg cells that secrete IL-10. It exerts its effects by inhibiting the synthesis of inflammatory cytokines and gene transcription, playing a significant role in finely tuning the Th17/Treg cells balance (Surana and Kasper, 2012). *Bifidobacterium breve* combined with non-digestible oligosaccharides can increase the secretion of IL-10 and the expression of Foxp3 in lung tissue, thereby enhancing the response of Tregs, reducing the activation of T lymphocytes and the expression of associated cytokines, and consequently lowering respiratory inflammatory responses (Sagar et al., 2014). One animal experiment found that Helicobacter pylori activates Treg cells directly through its neutrophil-activating protein, alleviating allergic airway disease in mice (Sehrawat et al., 2015). A key study has revealed that *Fecalibacterium prausnitzii* and *Akkermansia muciniphila* may promote the differentiation of Treg cells and enhance the production of the anti-inflammatory cytokine IL-10, while concurrently suppressing the secretion of pro-inflammatory cytokines such as IL-12. Notably, the abundance of these two beneficial microbial species was found to be significantly reduced in the gut microbiota of children with asthma (Demirci et al., 2019).

**Figure 2:**
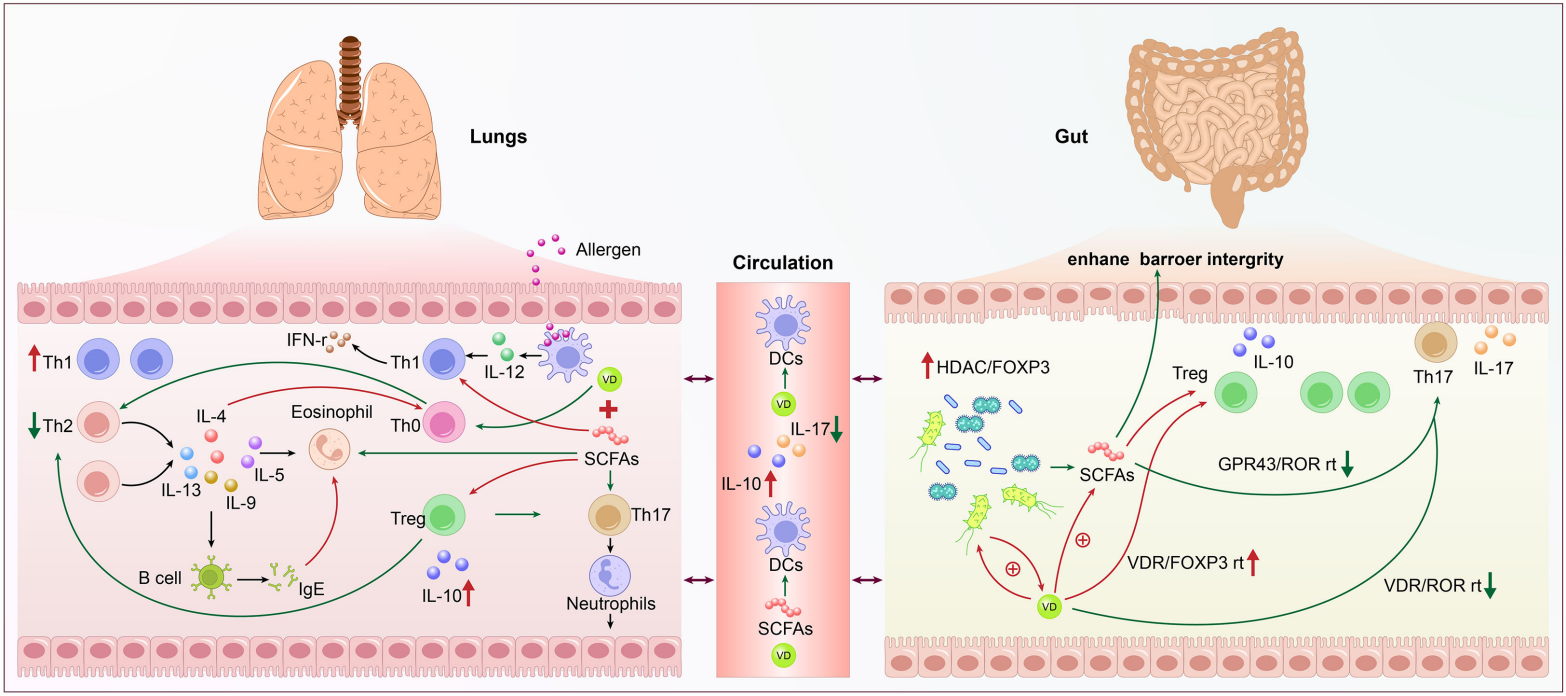
Schematic diagram of the immunometabolic mechanisms regulated by the gut-lung axis in asthma. The left side shows lung activity with allergens and various immune cells like Th1, Th2, Treg, eosinophils, and B cells interacting through cytokines such as IL-4, IL-5, IL-10, and IL-12. Th1 is increased, and Th2 is decreased. The right side details gut microbiome influence with Treg, Th17, and bacterial short-chain fatty acids (SCFAs) enhancing barrier integrity via pathways like VDR/FOXP3 and GPR43/ROR. Circulation links both systems with dendritic cells (DCs) influenced by SCFAs and Vitamin D. Components involve complex immune pathways and signaling molecules.

Although Th17 cells are crucial for preserving the integrity of the mucosal barrier and eliminating pathogens, a variety of inflammatory disorders are linked to their malfunction (Lee et al., 2020). In asthma, the activation of the Th17 axis regulates Th2 type inflammatory response in mice and humans, and is linked to a distinct asthma phenotype marked by low hormone responsiveness and neutrophil inflammation. Therefore, it may be a target for the treatment of severe asthma (Busse et al., 2013). The early growth and regulation of Th17 are significantly influenced by the gut microbiome. Many studies on the impact of microbiota on the Th17 axis have come from animal experiments. In mice, a type of Firmicutes, namely *segmented filamentous bacteria*, is sufficient to trigger the production of Th17 in the intestine, which can protect mice from further mucosal infections (Wang et al., 2019). The gut-lung axis is exemplified by the Th17 inflammatory response, in which intestine damage can result from Th17 responses brought on by respiratory infections and lung lesions from immune responses originating in the gut (Bradley et al., 2017). Supplementation of *Bifidobacterium longum* subspecies in human infants causes elevated indole lactic acid levels, thereby inhibiting Th17 and Th2 responses (Henrick et al., 2021). (**Figure 2**. Schematic Diagram of the Immunometabolic Mechanisms Regulated by the Gut-Lung Axis in Asthma.)

### 4.2 Imbalance of metabolic products

#### 4.2.1 Short-chain fatty acids

Resident bacteria in the gut that utilize dietary fiber to create SCFAs are considered key mediators of the gut-lung bidirectional interaction. The microbiota in the colon and cecum produces butyrate, propionate, and acetate, which are components of SCFA. They supply energy sources (particularly butyrate) and trigger a local immunological response in the stomach after being released into the lumen. SCFA is recognized by specific G protein-coupled receptors (GPCR) present on the surface of the intestinal tract and immune cells, such as GPCR109 or GPCR43. This interaction produces an antiallergenic response on the epithelial barrier and induces the differentiation of Treg cells in the colon (Macia et al., 2015). Depletion of these receptors disrupts the intestinal epithelial barrier in mice models, enhances allergen permeability and induces particular IgE responses (Tan et al., 2016). Because SCFA can boost macrophages and DC progenitor cells, which subsequently infiltrate the lungs and mature into CD11b+DC, whereas CD11b+DC is incapable of producing allergens, and downregulate hematopoiesis during Th2 allergic airway inflammation, they have a protective effect on allergic sensitization of the lungs (Kopf et al., 2015). In experiments, treatment of mice with high-and low-fiber diets found that circulating SCFA levels increased after a high-fiber diet, which suppressed type 2 inflammation and led to reduced airway hyperresponsiveness (Trompette et al., 2014). It has been demonstrated that SCFAs control systemic immune responses by blocking histone deacetylases or activating GPCRs. According to *Halnes* et al., SCFAs can reduce asthmatic airway inflammation by upregulating the gene expression of GPCRs, such as GPCR41 and GPCR43 (Halnes et al., 2017).

Among SCFA, butyrate is the most potent inhibitor of allergic immune responses in the lungs. This fatty acid inhibits Th2 and Th9 cell activation (Vieira et al., 2019),and induces Treg cell differentiation (Arpaia et al., 2013). Additionally, it prevents B cells’ IgE class turnover (Sanchez et al., 2020), migration of eosinophils to the airways and their chemotaxis, and induction of apoptosis of eosinophils (Theiler et al., 2019).*In vivo* studies of oral administration of butyrate have shown that butyrate induced asthma and weakened eosinophil infiltration in the lungs of mice (Roduit et al., 2019). Therefore, we believe that supplementation with dietary fiber can alleviate asthmatic inflammatory responses and ameliorate the condition. *Cait* et al. found that treatment with vancomycin can reduce the SCFA-producing gut microbiota, thus increasing susceptibility to ovalbumin-induced asthma and papain-induced lung inflammation in mice. Compared to controls, supplementation with short-chain fatty acids was found to reduce enhanced lung inflammation in vancomycin-induced asthmatic mice (Cait et al., 2018). According to a recent study, using Lactobacillus rhamnosus supplements can lower the prevalence of childhood asthma. This could be because the probiotic changes the microbiome’s makeup and SCFA levels (Chamitava et al., 2020). One study found that the lower the intake of fiber, the higher the chance of developing asthma (Saeed et al., 2020), while another study also found a positive correlation between fiber intake and improved lung function parameters. These studies all confirm that dietary fiber can effectively control asthma (Berthon et al., 2013). Therefore, it is expected to be a new way to treat or prevent asthma by increasing the level of SCFAs.

#### 4.2.2 Vitamin D

An increasing number of studies have found a significant correlation between VD deficiency and the onset, development, and prevention of childhood asthma (Uysalol et al., 2013; Uysalol et al., 2014). *Jones* et al. discovered that the gut microbiota can change VD metabolism and that probiotic supplements can influence VD levels based on the effect of gut microbiome dysbiosis on the development of asthma (Jones et al., 2013). VD has important immunomodulatory functions, capable of regulating the Th1/Th2 balance. VD can act on T cells, inhibiting the proliferation of Th2 cells and reducing total IgE levels, thereby suppressing the onset of asthma (Uysalol et al., 2013; Feketea et al., 2020). Additionally, VD can inhibit the differentiation and proliferation of dendritic cells, reducing the conversion of Th0 cells to Th2-type cells, thus indirectly inhibiting the development of chronic airway inflammation (Agrawal et al., 2013). Therefore, early monitoring of VD levels and supplementation with VD have significant implications for the prevention and management of asthma. (**Figure 2**. Schematic Diagram of the Immunometabolic Mechanisms Regulated by the Gut-Lung Axis in Asthma.).

### 4.3 Respiratory infections

Asthma is largely caused by respiratory virus infections, which can also cause severe flare-ups. Respiratory viral infections (such as rhinovirus, influenza virus, and respiratory syncytial virus) can alter the composition of both respiratory and gut microbiota, thereby influencing the development of asthma (Kloepfer and Kennedy, 2023). Changes in the gut microbiota caused by respiratory viral infections include an increase in opportunistic pathogens, a decrease in bacteria that produce SCFA, and a rise in *Bacteroidetes (* Groves et al., 2018; Woodall et al., 2022). These alterations are comparable to those seen in the gut microbiota of asthmatic people, indicating that an imbalance in the gut microbiota may play a role in the onset and exacerbation of asthma following a respiratory infection. *Wang* et al. were the first to investigate changes in the gut microbiota of asthmatic mice following respiratory syncytial virus (RSV) infection, demonstrating that RSV infection led to an increased abundance of *Bacteroidetes* and a decreased abundance of *Firmicutes phyla* in the intestinal microbiota of these mice (Wang et al., 2021).

The mechanism of asthma caused by the change of intestinal microorganisms after respiratory tract infection has not been fully understood. IFN-1 and IFN-3 are the main IFN subtypes produced by pulmonary viral infections, and IFNs are one of the inflammatory mediators produced during viral infections (Confino-Cohen et al., 2014). Previous studies have found that asthma patients may have impaired production of type I and type III IFN, thus increasing their susceptibility to viral infections. However, it has been found that type I interferons are promoted by SCFAs. Given the various functions of SCFAs previously discussed, it is thought that a reduction in advantageous SCFAs brought on by respiratory infections may lessen their ability to prevent asthma. By boosting interferon production, the body can better fight off respiratory viral infections and eradicate viruses, which will lessen the frequency of virus-induced asthma flare-ups (Zhao et al., 2023). Enhancing gut microbiota has been demonstrated to reduce viral loads and boost IFN-α and IFN-γ expression (Wang et al., 2021). It has been demonstrated that IFN-α decreases the quantity of infiltrating eosinophils in asthma patients’ airway tissue in a dose-dependent manner. This offers new possibilities for preventing virus-induced wheezing. We will explain the impact of the gut microbiota on the immune system and its direct or indirect relationship with the development of asthma.

## 5 Therapeutic strategies for asthma

Current clinical management of asthma primarily relies on long-term pharmacotherapy with corticosteroids, bronchodilators, and antihistamines. However, prolonged use of these agents is associated with specific adverse effects and the potential development of drug resistance. Based on the gut-lung axis theory, targeting the gut microbiota to modulate pulmonary immune responses represents a promising avenue for the prevention and treatment of asthma and other chronic respiratory diseases.

### 5.1 Probiotics

Probiotics have emerged as a key research focus in the adjunctive therapy of childhood asthma by modulating gut microbiota composition, restoring immune homeostasis, and enhancing gut-lung axis function.

Research indicates that probiotics may modulate asthma through three primary immunoregulatory mechanisms: First, probiotics systemically influence immune cell function and regulate inflammatory responses by producing short-chain fatty acids (SCFAs) such as acetate, butyrate, and propionate (Ratajczak et al., 2019). Second, probiotics modulate Toll-like receptors (TLRs) and activate dendritic cells (DCs), thereby enhancing Th1 cell responses. This process induces IFN-γ production by Th1 cells and natural killer (NK) cells, which subsequently suppresses Th2 cell activity and inhibits IgE formation in B cells, ultimately alleviating asthma symptoms (Idrees et al., 2022).Third, probiotics may beneficially impact asthma control by potentially altering specific microRNA expression profiles and improving lung function (Sadrifar et al., 2023).The most extensively studied probiotic strains in pediatric asthma primarily belong to the genera *Lactobacillus* and *Bifidobacterium*.

#### 5.1.1 Lactobacillus

Huang et al, taking *Lactobacillus* in asthmatics aged 6–18 years for three months, found improved control efficacy, increased peak expiratory flow, decreased IgE levels, and decreased asthma severity (Huang et al., 2018). *Lactobacillus* species, particularly *L. rhamnosus*, prevent airway hyperresponsiveness by reducing eosinophil infiltration and simultaneously suppressing type 2 inflammation (Spacova et al., 2020). To explore novel and possible therapeutic uses of probiotics in reducing asthma symptoms, researchers compared the long-term use of *L. rhamnosus* and *Bifidobacterium* breve to corticosteroid therapy. According to the study, *L. rhamnosus* was just as successful as budesonide in lowering airway resistance and the inflammatory response in the afflicted animals (Millares and Monso, 2022).Another recent animal study showed that the *Lactobacillus delbrueckii* can prevent the development of asthma in mice (Chen et al., 2024).

#### 5.1.2 Bifidobacterium

*Bifidobacterium* ameliorates asthma symptoms in induced murine models by upregulating inflammatory markers such as IFN-γ, IL-4, and IL-12, thereby modulating the balance between Th1 and Th2 responses (Mahooti et al., 2019; Wang et al., 2020).

#### 5.1.3 Other probiotic strains

Research has indicated that *C. butyricum* is a probiotic with significant development potential and has a certain therapeutic and preventive effect on bronchial asthma. *C. butyricum* can reverse the Th1/Th2 imbalance in asthmatic mice’s airways, boost the expression of the anti-inflammatory cytokine IL-10, and block the pulmonary NF-_k_B/NLRP3 inflammatory signaling pathway, further alleviating allergic airway inflammation (Juan et al., 2017; Li et al., 2022). *Kim* et al. recently found that oral administration of GTB1 can improve allergic airway inflammation in mice by producing butyrate (Kim et al., 2023).

#### 5.1.4 Limitations of probiotic interventions

While numerous studies highlight the potential benefits of probiotics in asthma management, it is essential to acknowledge conflicting evidence. For instance, a 2020 meta-analysis of 19 randomized controlled trials (n=5,157 children) concluded that probiotic supplementation was not associated with a reduced risk of asthma incidence in children compared to placebo (Wei et al., 2020).

Potential reasons for these neutral or negative findings include:Strain-Specificity: Effects are highly strain-dependent. Insufficiently screened strains may lack immunomodulatory properties.Timing and Dosage: Critical windows of intervention and optimal dosing regimens remain poorly defined. Host Microbiota Variability: Individual differences in baseline gut microbiota composition may influence probiotic colonization and efficacy. Methodological Heterogeneity: Variations in study design, asthma phenotypes, outcome measures, and probiotic formulations limit comparability across trials.

These limitations underscore the need for rigorous, phenotype-stratified trials and mechanistic studies to identify precise applications of probiotics in asthma management.

### 5.2 Short chain fatty acid supplementation

SCFAs are thought to have a significant role in the relationship between gut microbiota, food, and asthma development (Ríos-Covián et al., 2016). The fermentation of dietary fibers by the gut microbiota produces SCFAs, mainly acetate, propionate, and butyrate. These metabolites are a promising therapeutic target for the treatment of asthma since they have been demonstrated to have a major impact on immune system modulation and may aid in reducing pro-inflammatory responses in the lungs (Barcik et al., 2020). Supplementation with SCFAs or a high-fiber diet that promotes high SCFA production has shown beneficial effects in allergic airway diseases. Huang et al. ‘s latest study found that depletion of polymorphonuclear myeloid suppressor cells eliminated the protective effect of SCFA in allergic airway inflammation, so supplementation of SCFA could enhance the differentiation of PMN-MDSCs, thereby improving airway allergic response (Huang et al., 2023). Many studies have shown that populations with the highest intake of dietary fiber have the lowest rates of allergic asthma (Saeed et al., 2020; Lee et al., 2021). Combining the pathogenic mechanisms and existing research findings, In addition to being a potential therapeutic target for allergic inflammatory disorders in other organs, SCFAs may also be a therapeutic target for pulmonary allergic inflammatory diseases.

### 5.3 Fecal microbiota transplantation

Fecal Microbiota Transplantation (FMT) is a therapeutic approach aimed at reconstructing gut microbial diversity and suppressing the proliferation of harmful bacteria in the intestines. This is achieved by transferring specially processed microbiota from the feces of healthy donors into the gastrointestinal tract of patients (He et al., 2023). Currently, the only formally approved indication for FMT is recurrent Clostridioides difficile infection (rCDI) (Ooijevaar et al., 2019). For other conditions, such as inflammatory bowel disease, irritable bowel syndrome, hepatic encephalopathy, and metabolic syndrome, FMT remains in the research and exploratory stages. The “gut-lung axis” theory suggests that FMT may hold significant therapeutic potential for asthma. Recent animal studies have found that asthma induction led to gut microbiota dysbiosis in rats. FMT treatment ameliorated the inflammatory response in these asthmatic rat models and corrected their disruptions in gut short-chain fatty acids (SCFAs) (Lai et al., 2025). Furthermore, after FMT restored the gut microbiota in asthmatic mice, the respiratory microbiota dysbiosis was partially improved, and airway inflammation was significantly alleviated (Zheng et al., 2025).

It must be pointed out, however, that the use of FMT carries a certain risk of pathogen contamination, which may increase the incidence of immune-related adverse events. Currently, there is a limited number of animal experimental studies on FMT for asthma treatment, and no human clinical research data have been disclosed yet. Therefore, it is imperative to adopt stringent safety strategies to ensure the safety and efficacy of FMT. These include rigorous quality control and supervision, encompassing donor screening, standardized stool processing, pre-transplantation microbial composition analysis, post-transplant monitoring, and long-term follow-up to assess sustained efficacy and safety.

### 5.4 Phytogenic compounds

Phytogenic compounds have increasingly garnered research interest for their multi-targeted interventions in asthma with minimal side effects, primarily through modulation of the gut microbiota and immunoregulatory effects. For instance, the polyphenol curcumin has been demonstrated to suppress the proliferation, migration, and inflammatory infiltration of airway smooth muscle cells in murine asthma models. It also alters the composition of the gut microbiome in asthmatic mice and ameliorates Th2-mediated inflammatory responses, thereby exerting therapeutic effects against asthma (Wu et al., 2021; Ma et al., 2024). Additionally, Sodium houttuyfonate (SH), an active compound derived from Houttuynia cordata Thunb, alleviates asthma symptoms by rebalancing Th1/Th2 immunity and modulating the gut microbiome (Guo et al., 2024). Daqing Formula (DQF), composed of the leaves and roots of the plant *Isatis indigotica* Fort, has been shown to significantly reduce pulmonary inflammation in murine models. It ameliorates the lung microbiome, inhibits the growth of multiple pathogenic bacteria, and concurrently modulates levels of inflammatory cytokines. Furthermore, DQF restores the Th1/Th2 cell balance and suppresses the activation of eosinophils. These results collectively indicate that DQF holds promising potential as a therapeutic intervention for severe asthma (Wu et al., 2024). However, asthma pathogenesis involves a complex interplay of immune, neural, and environmental factors. To address such multidimensional complexity, Traditional Chinese Medicine (TCM) have long employed multi-herb formulations that leverage synergistic interactions among numerous botanical constituents to achieve broader and more potent therapeutic effects than single compounds. For instance:Tuo-Min-Ding-Chuan Decoction (TMDC), is a traditional Chinese herbal formulation composed of twelve distinct botanical medicinal components. Studies in murine models of asthma have demonstrated that TMDC significantly alleviates pulmonary inflammation and eosinophilic infiltration, reduces the secretion of the pro-inflammatory cytokines IL-4 and IL-5, and promotes an increase in regulatory Treg cell populations within the lungs, small intestine, and colon. Furthermore, TMDC administration has been shown to restore gut microbial diversity and enhance the abundance of beneficial bacteria, notably Bifidobacterium and Lactobacillus. This multi-faceted action, targeting both immunomodulation and microbiome restoration, correlates with its excellent efficacy in alleviating clinical symptoms and reducing the frequency of asthma exacerbations (Zhou et al., 2022; Hong et al., 2025).

## 6 Human vs. animal studies in asthma and gut microbiota

The references cited in this article regarding the relationship between gut microbiota and asthma, as identified in both human and animal studies, have been compiled and are presented in **Table 1** Human vs. Animal Studies in Asthma and Gut Microbiota.

**Table 1:** Human vs. animal studies in asthma and gut microbiota.

Study population	Microbiota	Findings	References
High-risk infants (for asthma)	Lachnospira, Veillonella, Fecalibacterium, Rothia	Relative abundance decreased	(Wang et al., 2020)
Born-immune newborn cohort study	Bifidobacterium longum	The supplementation causes elevated indole lactic acid levels, inhibiting Th17 and Th2 responses	(Henrick et al., 2021)
Children with asthma	Bifidobacterium longum	Lower detection rate	(Akay et al., 2014)
	Lactobacillus, Bifidobacterium	Oral supplements relieve asthma symptoms	(Huang et al., 2018; Chamitava et al., 2020; Yang et al., 2023)
	Prevotella copri, Bifidobacterium breve, Bifidobacterium catenulatum	Relative abundance decreased	(Mahdavinia et al., 2023)
	L. rhamnosus, L. casei, Bifidobacterium brevis	Oral supplements relieve asthma symptoms	(Shi et al., 2021)
	Fecalibacterium prausnitzii, Akkermansia muciniphila	Relative abundance decreased	(Demirci et al., 2019)
Mouse model	Inoculate germ-free mice with Lachnospira, Veillonella, Fecalibacterium, Rothia	Reduced airway inflammation	(Arrieta et al., . 2015)
	Isprevalenzia, Brauneria, Marvinbryantia	Following exposure to cefixime, the genera were enriched.	(Kong et al., 2024)
	L. rhamnosus, L. casei, Bifidobacterium brevis	Oral supplements relieve asthma symptoms	(Mahooti et al., 2019; Spacova et al., 2020; Wang et al., 2020; Shi et al., 2021)
	L. johnsonii	The supplementation reduced the Th2 response, decreased both allergy and airway hyperresponsiveness	(Fujimura et al., 2014)
	L.reuteri	Alleviates airway inflammation, reduces total IgE levels, decreases Th2-associated proinflammatory cytokines	(Li et al., 2020b)
	Bacteroides fragilis	PSA can induce the differentiation of CD4+ T cells into Treg cells that secrete IL-10, playing a significant role in the Th17/Treg cells balance	(Surana and Kasper, 2012)
	Bifidobacterium breve	Combined with non-digestible oligosaccharides, it elevates levels of IL-10 and Foxp3 in the lungs and attenuates inflammation	(Sagar et al., 2014)
	Helicobacter pylori	Asthma was significantly reduced in newborn mice after infection	(Sehrawat et al., 2015)
	segmented filamentous bacteria	Promotes the differentiation of Th17 cells in the gut	(Wang et al., 2019)
	Bacteroidetes Firmicutes phyla	After RSV infection in asthmatic mice, the Bacteroidetes increased while the Firmicutes phylum decreased in the gut microbiota	(Wang et al., 2021)
	Lactobacillus delbrueckii	Prevent the development of asthma	(Chen et al., 2024)
	C. butyricum	Can reverse the Th1/Th2 imbalance, boost the expression of IL-10, alleviating allergic airway inflammation	(Juan et al., 2017; Li et al., 2022)

## 7 Clinical trials registry

In recent years, there has been a growing number of clinical studies on asthma based on the “gut-lung axis” theory. To investigate current clinical research trends, we conducted searches in the Chinese Clinical Trial Registry.

(ChiCTR), ClinicalTrials.gov, and the European Union Clinical Trials Register (EudraCT) using the search terms: (“asthma”) AND (“gut microbiota” OR “gut-lung axis”), with a date range from January 1, 2020, to March 2025. The search in the ChiCTR identified 216 asthma-related clinical studies, among which 6 were associated with gut microbiota. The research focus of these trials is summarized in **Table 2**. On ClinicalTrials.gov, 1,205 studies with asthma as the primary topic were retrieved, 18 of which were related to gut microbiota, including both interventional and observational studies. Their research content is also detailed in **Table 2**. In contrast, no asthma trials associated with gut microbiota were found among the 201 asthma-related studies in EudraCT.

**Table 2:** Registry studies: gut microbiota and asthma.

Study focus	ChiCTR	Clinicaltrials.gov
interventional studies	4	9
observational studies	2	9
Gut flora and metabolites	1	7
Vitamin D-Gut Microbiota Interaction	0	3
Early-Life Cohort Study	0	3
Traditional Chinese Medicine	3	0
Probiotics	1	3
Antibiotics	0	1
Environmental Intervention	0	1
Helicobacter pylori	1	0

A possible reason for the absence of such studies in EudraCT is that researchers may have chosen to register their trials on ClinicalTrials.gov instead, as several gut microbiota-asthma studies conducted by investigators from China and Europe were identified on that platform. Therefore, **Table 2** summarizes the research content based on registrations in both the Chinese Clinical Trial Registry and ClinicalTrials.gov.

From the study data presented in **Table 2**, we synthesized the current trends in clinical research linking gut microbiota and asthma:

### 7.1 Gut microbiota and metabolites

There is growing interest in characterizing the composition of gut microbiota and microbiota-derived metabolites in patients with asthma. Researchers are increasingly employing multi-omics approaches to identify potential biomarkers and elucidate underlying mechanisms.

### 7.2 Vitamin D–gut microbiota interaction

This remains an active area of investigation, particularly concerning how.

vitamin D status and supplementation may interact with gut microbial communities to modulate asthma risk and severity across different age groups, including maternal-child cohorts.

### 7.3 Early-life cohort studies

Prospective birth and early-life cohort studies remain essential for understanding the developmental origins of asthma. Current research focuses on how early microbial exposures, diet, antibiotic use, and environmental factors influence immune development and long-term respiratory outcomes.

### 7.4 Traditional Chinese medicine

Chinese researchers have shown growing interest in exploring the potential of herbal formulations, massage techniques, and acupoint applications in modulating gut microbiota and improving asthma control. These efforts aim to integrate traditional Chinese medicine with modern immunomodulatory concepts through evidence-based research, thereby providing scientific validation of the TCM theory that “the lung stands in interior-exterior relationship with the large intestine”.

### 7.5 Probiotics

Research continues to explore specific probiotic strains and synbiotic combinations for the primary prevention and adjunctive management of asthma. Emphasis is placed on strain-specific effects, timing of intervention (e.g., prenatal vs. early childhood), and personalized microbial therapeutics.

According to the latest registry updates, no results have been uploaded yet for clinical studies corresponding to these areas. The absence of published outcomes underscores the novelty of these research directions. These trials are either ongoing or nearing completion, and their findings are highly anticipated to provide valuable insights into mechanistic pathways and clinical applications related to gut microbiota and asthma.

However, these clinical studies still have several limitations that warrant careful consideration in both their design and interpretation: Firstly, issues related to sample size and population heterogeneity are prominent. Most trials have limited sample sizes, and significant variations exist among populations in terms of asthma phenotypes, baseline gut microbiota, genetic background, geographical environment, and dietary habits. These differences may limit the generalizability of the findings to broader asthma populations. Secondly, there is a lack of standardization in intervention protocols. Considerable disparities exist across studies in the strains of probiotics used, dosages, and duration of interventions. The absence of unified intervention standards makes it difficult to directly compare outcomes between studies. Lastly, data on long-term efficacy and safety are insufficient. Most trials focus on short-term effects, while the modulation of gut microbiota and its potential impact on asthma may require extended observation periods.

## 8 Discussion

Asthma is a highly prevalent chronic respiratory disease that poses a significant burden in the pediatric population. It is characterized by recurrent exacerbations, and a subset of children exhibit suboptimal long-term control with conventional pharmacotherapy. This review synthesizes emerging evidence underscoring the pivotal role of gut microbiota in the immunopathogenesis and potential management of asthma.

Accumulating findings indicate profound interactions among the gut microbiota, host immunity, and respiratory health, conceptualized through the gut–lung axis. Advances in microbiome science have elucidated how microbial communities and their metabolites influence asthma pathogenesis and progression, opening new avenues for therapeutic intervention. Proposed strategies include probiotic administration, targeted modulation of microbial metabolites, and fecal microbiota transplantation (FMT), reflecting a paradigm shift toward host–microbe crosstalk as a central element in asthma management.

However, translation of these findings into clinical practice warrants cautious interpretation. The current evidence is derived from a heterogeneous body of animal studies, observational human research, and a limited number of randomized controlled trials (RCTs), each with inherent methodological limitations. Much of the mechanistic insight discussed in this review originates from murine models. While invaluable for delineating pathophysiological pathways, these are constrained by interspecies differences in immune function and microbiome architecture. Cross-sectional and case-control studies offer suggestive associations but remain susceptible to confounding variables such as diet, environmental exposures, and medication use. Prospective cohort studies and RCTs provide the most robust evidence; future efforts should prioritize larger, multicenter trials with standardized designs, integrated with systems biology approaches to unravel intricate microbiota–host interactions.

Although this review constructs a framework linking gut microbiota and asthma, unaddressed biases may affect the reliability of conclusions. Notably, positive outcomes are overrepresented in the literature, while negative or neutral results, particularly from small-scale studies are underreported and less likely to be published.

In summary, current research highlights the promising role of gut microbiota as a therapeutic target in asthma, yet significant challenges remain in study design, mechanistic validation, and clinical translation. To address these limitations, future investigations should focus on three pillars: standardization of methodologies, precision in experimental design, and in-depth mechanistic exploration.

## 9 Conclusion

Overall, clinical research on pediatric asthma and gut microbiota is evolving from associative observations toward mechanistic inquiry and targeted interventions. Efforts are increasingly directed at deciphering the complexities of gut–lung communication and developing microbiota-based strategies for asthma prevention and treatment. Despite existing challenges, this field offers novel directions and hope for comprehensive asthma management, particularly for children with inadequate response to conventional therapies or those seeking alternative treatment options.
